# Supplementary material for: STAG2 deficiency induces interferon responses via cGAS-STING pathway and restricts virus infection
Source: Nat Commun. 2018 Apr 16;9:1485. doi: 10.1038/s41467-018-03782-z (PMC5902600; doi:10.1038/s41467-018-03782-z)
Supplement: Supplementary file 1 — Supplementary Information [file 41467_2018_3782_MOESM1_ESM.pdf]

Supplementary Information for

STAG2 deficiency induces interferon responses via cGAS-STING pathway and restricts virus infection

Corresponding author: Harry B. Greenberg, hbgreen@stanford.edu

Supplementary Figures and Figure Legends

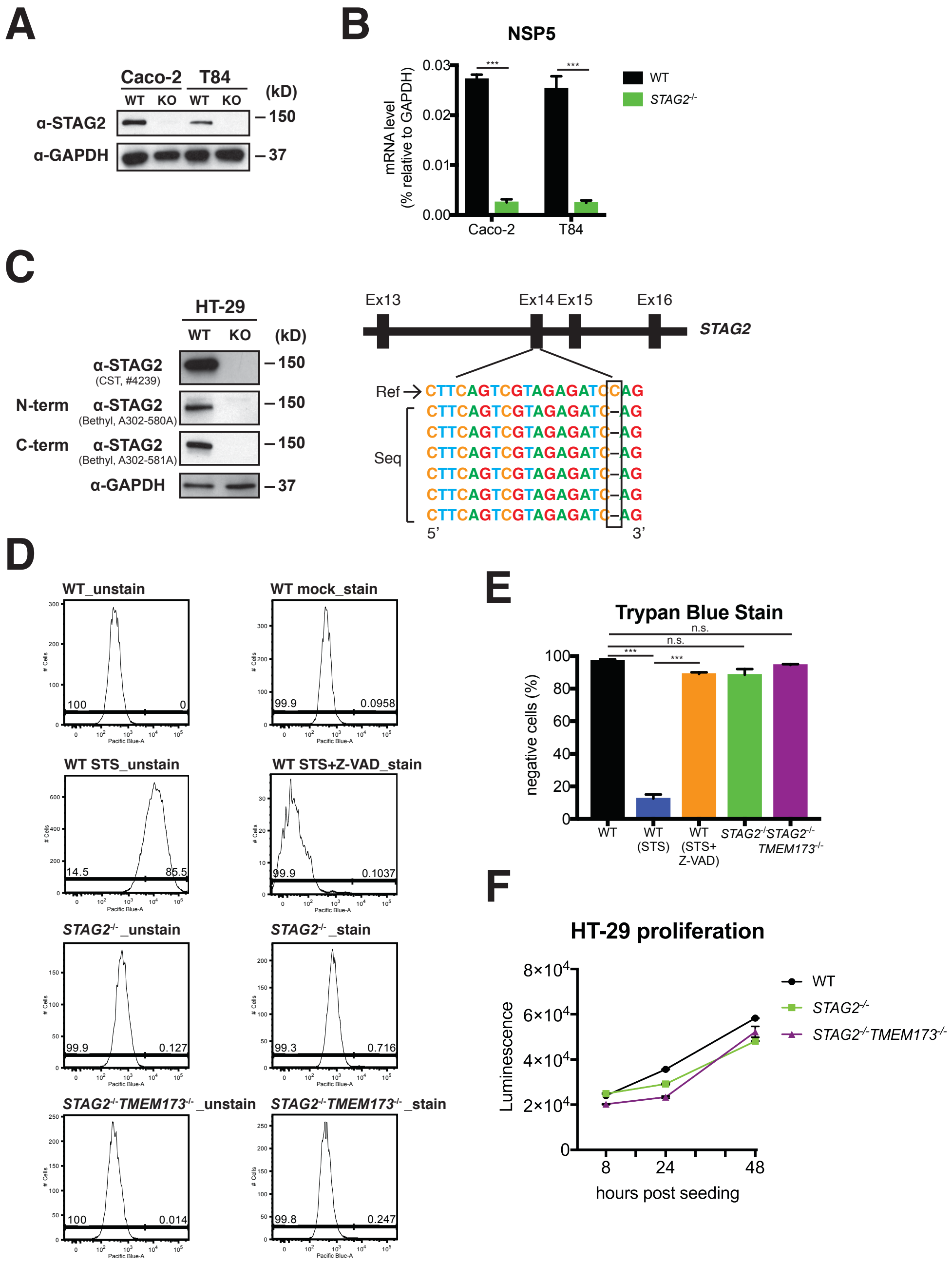

Supplementary Figure 1. STAG2 deficiency results in decreased RV replication

(**A**) Caco-2 and T84 cells were transduced with lentiviruses encoding Cas9 and STAG2 sgRNA, cultured for 14 days under puromycin selection (3 μg/ml and 6 μg/ml respectively) and lysates were analyzed by western blot using indicated antibodies. (**B**)

Cells in **A** were infected with bovine RV NCDV strain (MOI=1) and viral NSP5 mRNA level was measured at 24 h.p.i. by RT-qPCR. (**C**) Validation of clonal STAG2 knockout HT-29 cells by western blot using three different antibodies targeting different epitopes of STAG2 (left panel) and Sanger sequencing (right panel). (**D** and **E**) WT HT-29 cells were treated with 1 μM staurosporine (STS) with or without 10 μM Z-VAD-FMK (Z-VAD) for 24 hr. WT and KO HT-29 cells were subject to live/dead staining (Pacific Blue, numbers from left to right indicating percentage of live and dead cells) by flow cytometry (**D**) or trypan blue staining (**E**). (**F**) WT and KO HT-29 cell numbers were measured by a RealTime-Glo Cell Viability Assay at indicated time points. For all figures, experiments were repeated at least three times in triplicates. Data are represented as mean ± SEM.

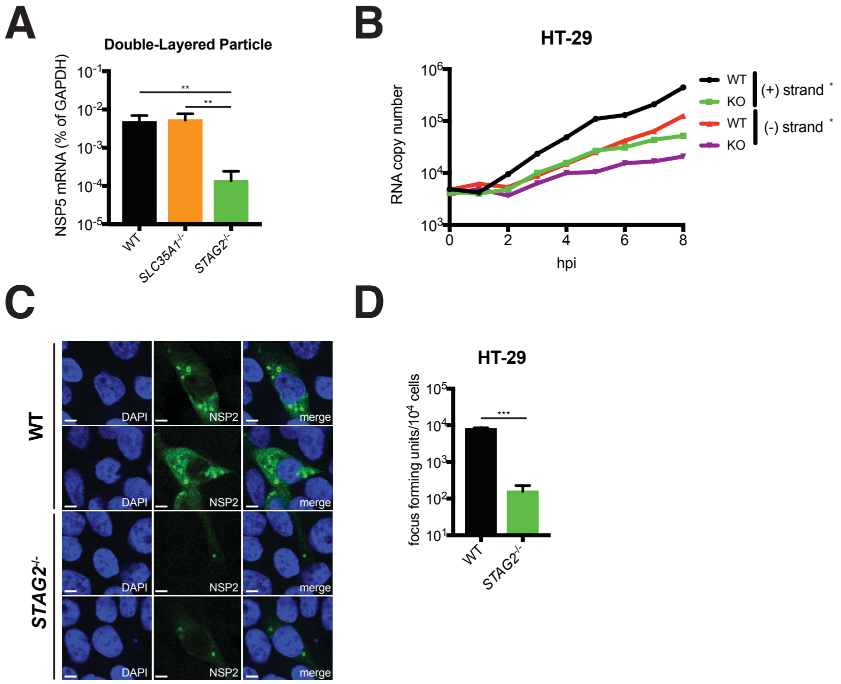

Supplementary Figure 2. Multiple RV replication cycle steps were suppressed in the absence of STAG2

(**A**) Wild-type (WT), *SLC35A1*$^{-/-}$, *STAG2*$^{-/-}$ HT-29 cells were transfected with bovine RV UK strain double-layered particle (DLP) using lipofectamine [1] to bypass entry and directly deliver viral RNA into the cytoplasm. Viral NSP5 mRNA level was measured at 16 hr post transfection by RT-qPCR. (**B**) WT and *STAG2*$^{-/-}$ HT-29 cells were infected with UK (MOI=1) and total RNA was harvested at every hour post infection for strand-specific RT-qPCR measuring the positive (+) and negative (-) strands of RV gene segment 7. (**C**) WT and *STAG2*$^{-/-}$ HT-29 cells were infected with UK (MOI=1) for 24 hr and were analyzed by immunofluorescence: nucleus (DAPI, blue) and NSP2 (mAb, clone 191, green). Scale bar: 5 μM. (**D**) WT and *STAG2*$^{-/-}$ HT-29 cells were infected with UK (MOI=1). Supernatants were harvested at 24 h.p.i. and virus yield was measured by a

standard plaque assay [2]. For all figures, experiments were repeated at least three times in triplicates. Data are represented as mean ± SEM.

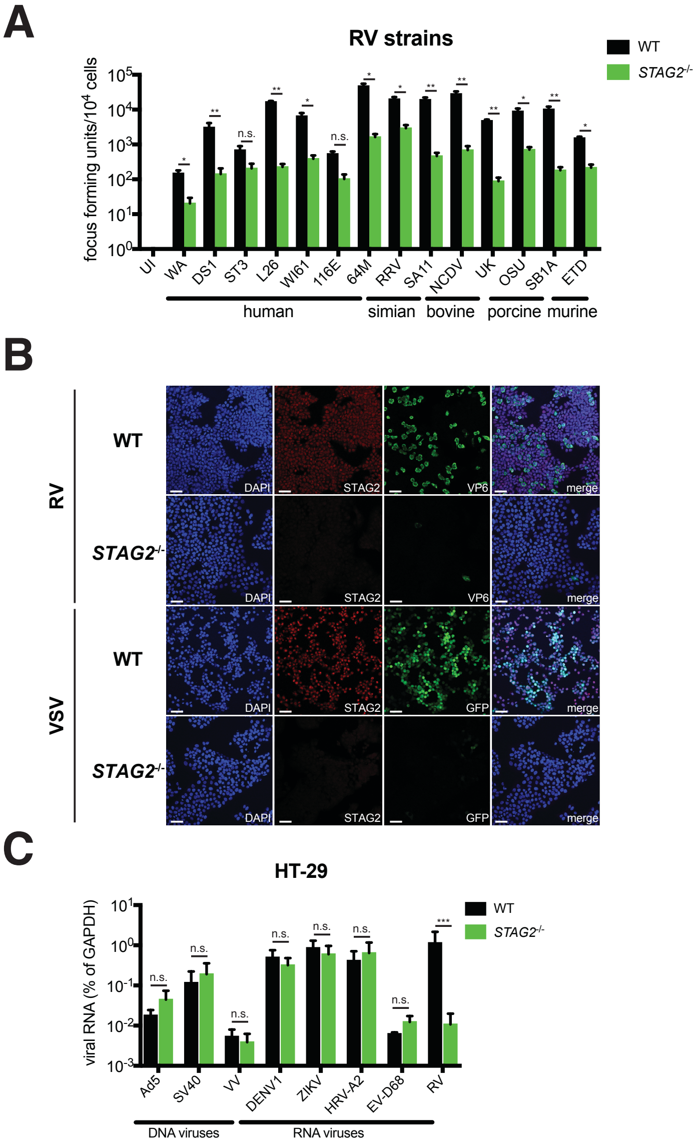

Supplementary Figure 3. STAG2 deletion inhibits the replication of IFN-sensitive RNA viruses

(**A**) WT and *STAG2*$^{-/-}$ HT-29 cells were infected with indicated human and animal RV strains (MOI=1) for 24 h.p.i. and virus yield in the supernatant was measured by a standard plaque assay. Wa: G1, P[8]; DS1: G2, P[4]; ST3: G4, P[6]; L26: G12, P[4]; WI61: G9, P[8]; 116E: G9, P[11]; 64M: G8, P[10]; RRV: G3, P[3]; SA11-4F: G3, P[1];

NCDV: G6, P[1]; UK: G6, P[5]; OSU: G5, P[7]; SB1A: G4, P[6]; ETD: G16, P[16]. (**B**) WT and *STAG2*$^{-/-}$ HT-29 cells were infected with RV (bovine UK strain) or VSV at MOI of 1 for 16 hr and analyzed by immunofluorescence: nucleus (DAPI, blue), STAG2 (red), and viral antigens (VP6 for RV; GFP for VSV, green). Scale bar: 50 μM. (**C**) WT and *STAG2*$^{-/-}$ HT-29 cells were infected with indicated DNA or RNA viruses (MOI=1) for 24 h.p.i. and viral RNA levels were measured by RT-qPCR. Ad5: human adenovirus serotype 5; SV40: simian vacuolating virus 40; VV: vaccinia virus; DENV1: dengue virus serotype 1; ZIKV: Zika virus; HRV: human rhinovirus; EV: enterovirus; RV: rotavirus. For all figures, experiments were repeated at least three times in triplicates. Data are represented as mean ± SEM.

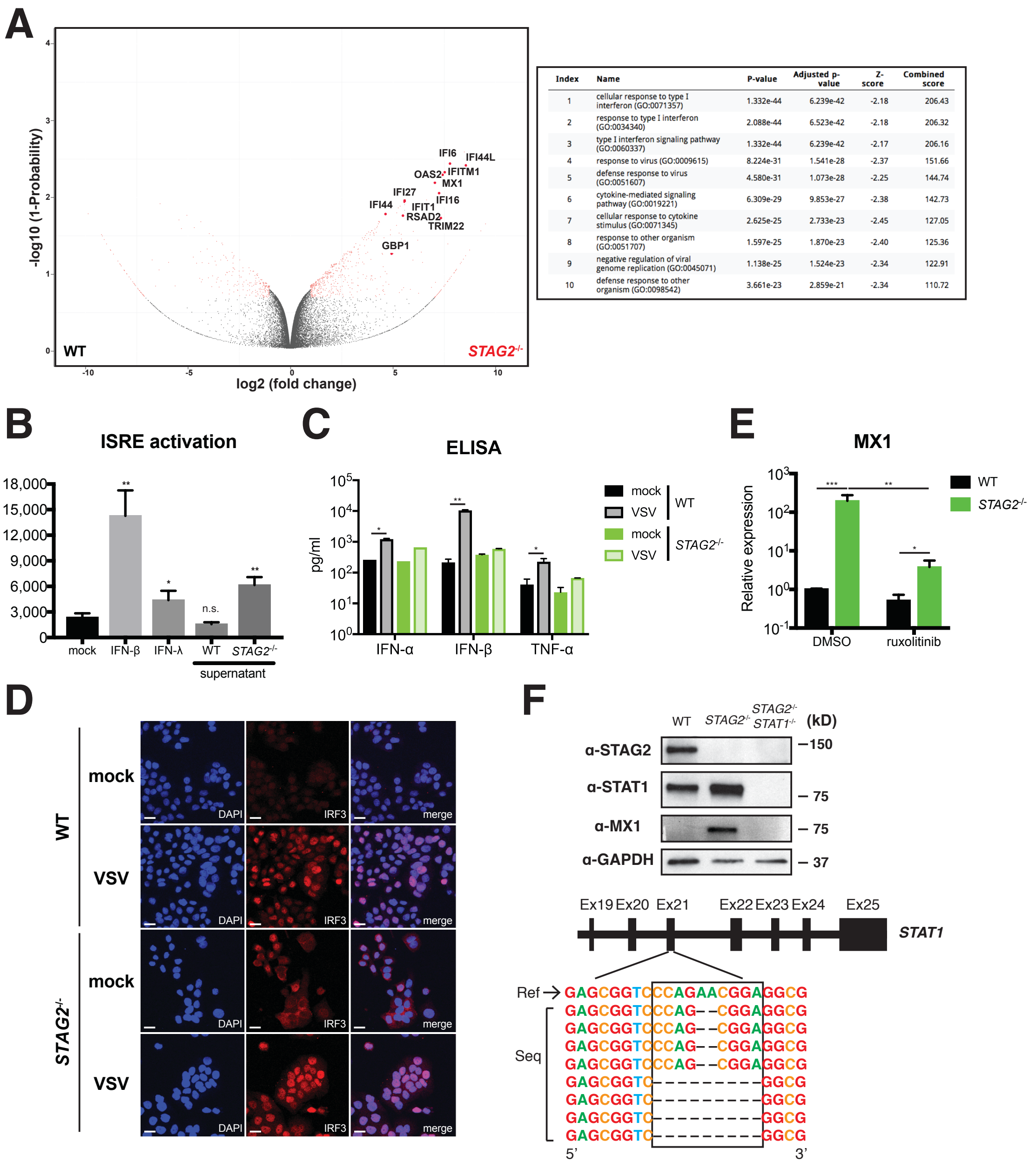

Index	Name	P-value	Adjusted p-value	Z-score	Combined score
1	cellular response to type I interferon (GO:0071357)	1.332e-44	6.239e-42	-2.18	206.43
2	response to type I interferon (GO:0034340)	2.088e-44	6.523e-42	-2.18	206.32
3	type I interferon signaling pathway (GO:0060337)	1.332e-44	6.239e-42	-2.17	206.16
4	response to virus (GO:0009615)	8.224e-31	1.541e-28	-2.37	151.66
5	defense response to virus (GO:0051607)	4.580e-31	1.073e-28	-2.25	144.74
6	cytokine-mediated signaling pathway (GO:0019221)	6.309e-29	9.853e-27	-2.38	142.73
7	cellular response to cytokine stimulus (GO:0071345)	2.625e-25	2.733e-23	-2.45	127.05
8	response to other organism (GO:0051707)	1.597e-25	1.870e-23	-2.40	125.36
9	negative regulation of viral genome replication (GO:0045071)	1.138e-25	1.524e-23	-2.34	122.91
10	defense response to other organism (GO:0098542)	3.661e-23	2.859e-21	-2.34	110.72

Supplementary Figure 4. Loss of STAG2 leads to a spontaneous IFN activation

(**A**) Volcano plot of RNA-sequencing data (Illumina system) from uninfected WT and *STAG2*$^{-/-}$ HT-29 cells (left panel); Gene ontology analysis of biological pathways associated with RNA-sequencing data from Fig. 2B and Fig. S4A (right panel). (**B**) Cell culture medium of WT and *STAG2*$^{-/-}$ HT-29 cells were collected and added to HT-29 cells that express an ISRE promoter driven firefly luciferase (FFL) and CMV promoter

driven renilla luciferase (RL) as an internal control. IFN-β (100 U/ml) and IFN-λ (100 ng/ml) were used as positive controls. (**C**) IFN-α, IFN-β, and TNF-α secretion was measured by ELISA in mock or VSV-infected WT and *STAG2*$^{-/-}$ HT-29 cells. (**D**) WT and *STAG2*$^{-/-}$ HT-29 cells were infected with VSV (MOI=5) for 8 hr and analyzed by immunofluorescence: nucleus (DAPI, blue) and IRF3 (red). Scale bar: 20 μM. (**E**) WT and *STAG2*$^{-/-}$ HT-29 cells were treated with vehicle control DMSO or JAK inhibitor ruxolitinib (100 nM) for 24 hr. Total RNA was harvested for RT-qPCR measuring MX1 mRNA level, normalized to that of GAPDH. (**F**) Validation of clonal STAG2, STAT1 double knockout HT-29 cells with western blot (upper panel) and Sanger sequencing (lower panel). For all figures except (a), experiments were repeated at least three times in triplicates. Experiments of (a) were performed in duplicates on two different sequencing platforms. Data are represented as mean ± SEM.

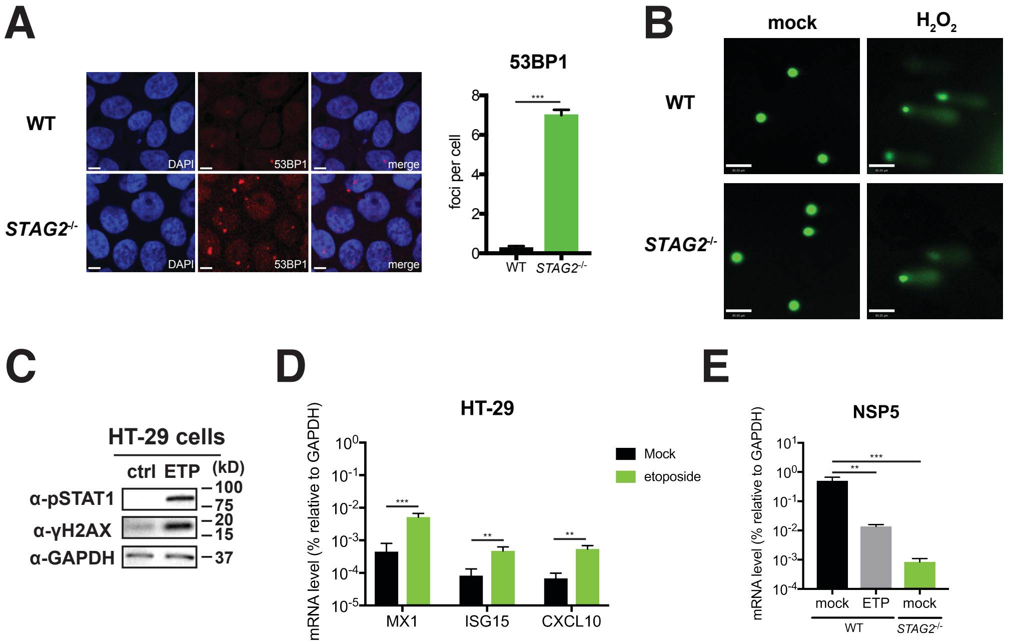

Supplementary Figure 5. Increased double-stranded DNA breaks were observed upon STAG2 depletion

(**A**) WT and *STAG2*$^{-/-}$ HT-29 cells were analyzed by immunofluorescence: nucleus (DAPI, blue) and 53BP1 (red). Scale bar: 3.5 µm. 53BP1 puncta number was quantified by ImageJ. (**B**) WT or *STAG2*$^{-/-}$ HT-29 cells were treated with 100 µM hydrogen peroxide for 20 minutes and analyzed by single-cell electrophoresis. Scale bar: 80 µM. (**C**) HT-29 cells were treated with 10 µM etoposide (ETP) for 24 hr and harvested for western blot analysis. (**D**) Same as (**C**) except that total RNA was harvested for RT-qPCR measuring indicated ISG mRNA level, normalized to that of GAPDH. (**E**) Mock or ETP-treated WT HT-29 cells and *STAG2*$^{-/-}$ HT-29 cells were infected with RV (MOI=1) for 24 hr and examined by RT-qPCR for viral NSP5 levels. For all figures, experiments were repeated at least three times in triplicates. Data are represented as mean ± SEM.

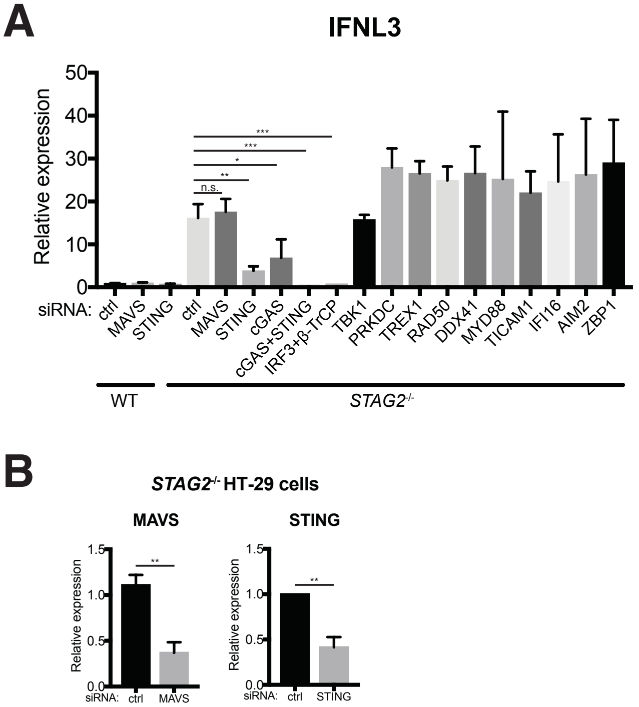

Supplementary Figure 6. cGAS and STING mediate IFN induction in STAG2 deficient cells

(**A**) WT and *STAG2*$^{-/-}$ HT-29 cells were transfected with indicated siRNA for 48 hr. Total RNA was then harvested and analyzed by RT-qPCR for IFNL3 mRNA level, normalized to that of GAPDH. (**B**) mRNA levels of MAVS (left panel) and STING (right panel) in siRNA-transfected *STAG2*$^{-/-}$ HT-29 cells, normalized to that of GAPDH. For all figures, experiments were repeated at least three times in triplicates. Data are represented as mean ± SEM.

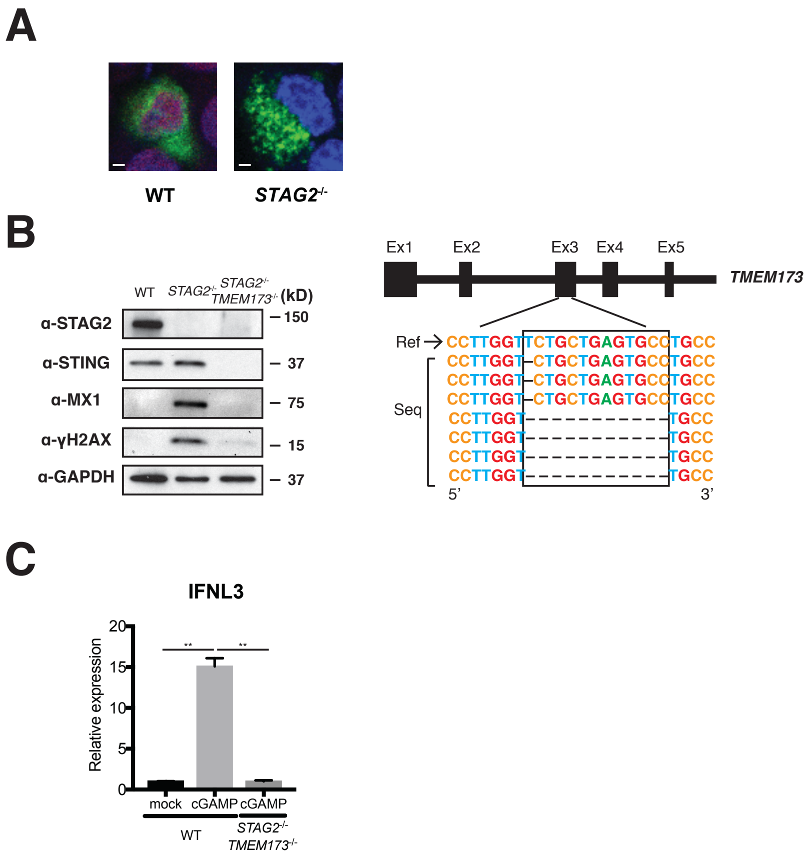

Supplementary Figure 7. STING is responsible for IFN activation in STAG2 deficient cells

(**A**) High-resolution confocal images of WT and *STAG2*$^{-/-}$ HT-29 cell stably expressing HA-STING: nucleus (DAPI, blue), STAG2 (red) and STING (HA, green). Scale bar: 1 μM. (**B**) Validation of clonal STAG2, STING double knockout HT-29 cells with western blot (left panel) and Sanger sequencing (right panel). (**C**) WT and *STAG2*$^{-/-}$*STING*$^{-/-}$ HT-29 cells were transfected with cGAMP (8 μg/ml) for 8 hr. Total RNA was then harvested and analyzed by RT-qPCR for IFNL3 mRNA level, normalized to that of GAPDH. For all figures, experiments were repeated at least three times in triplicates. Data are represented as mean ± SEM.

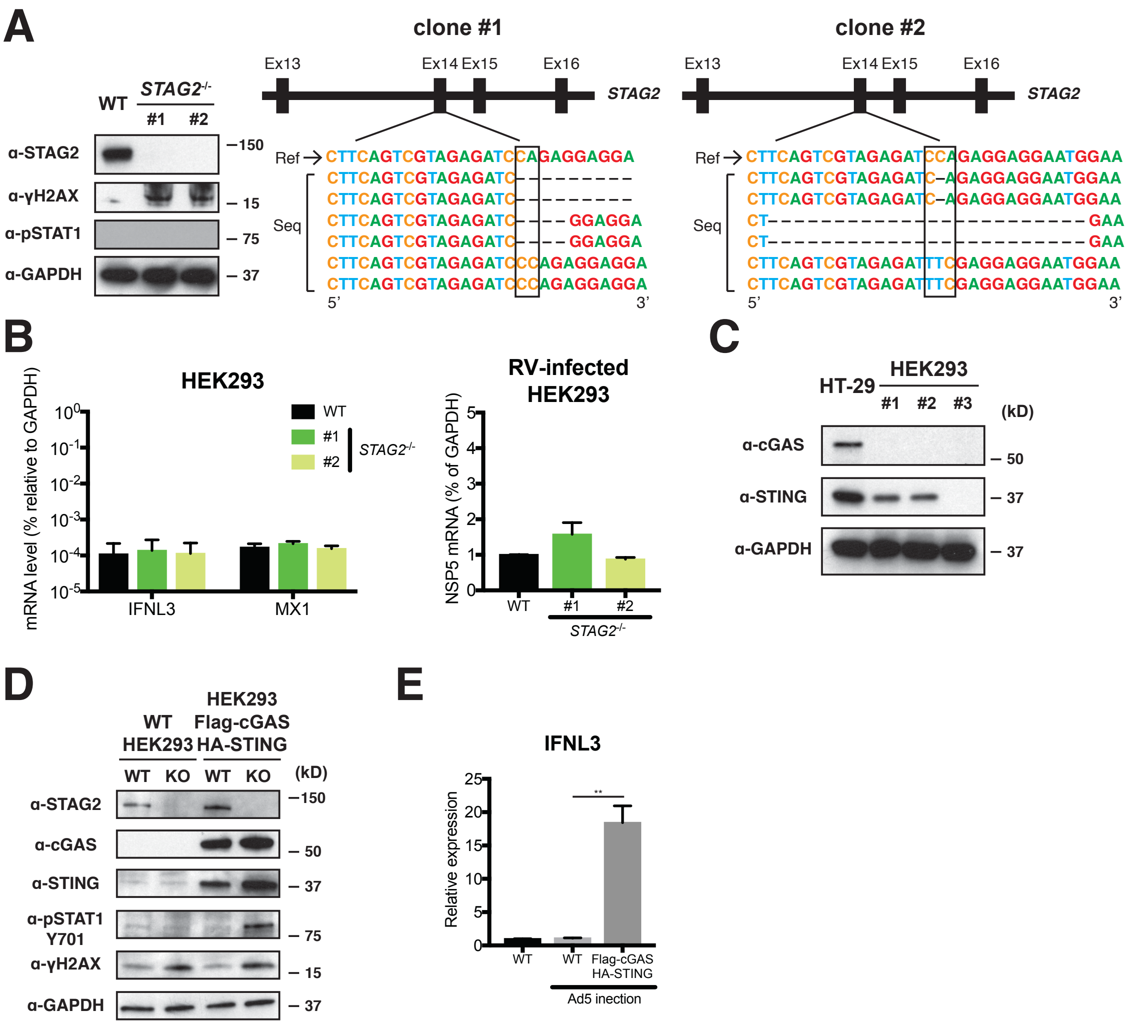

Supplementary Figure 8. HEK293 cells fail to activate IFN expression upon STAG2 deletion

(**A**) Validation of two individual clonal STAG2 knockout HEK293 cells with western blot (left panel) and Sanger sequencing (right panel). (**B**) Expression of IFNL3 and MX1 were measured in uninfected WT and *STAG2*$^{-/-}$ HEK293 cells and compared to that of GAPDH (left panel). WT and *STAG2*$^{-/-}$ HEK293 cells were infected with RV (MOI=1) for 24 h.p.i. and viral NSP5 mRNA level was measured by RT-qPCR (right panel). (**C**) Lysates of HT-29 cells and HEK293 cells (from three different laboratories) were analyzed by western blot using indicated antibodies. (**D**) Lysates of WT and *STAG2*$^{-/-}$

HEK293 cells, mock or stably expressing Flag-cGAS and HA-STING were analyzed by western blot using indicated antibodies. (**E**) WT HEK293 cells, mock or stably expressing Flag-cGAS and HA-STING, were infected with adenovirus serotype 5 (MOI=5) and total RNA was harvested at 8 h.p.i. for RT-qPCR analysis. For all figures, experiments were repeated at least three times in triplicates. Data are represented as mean ± SEM.

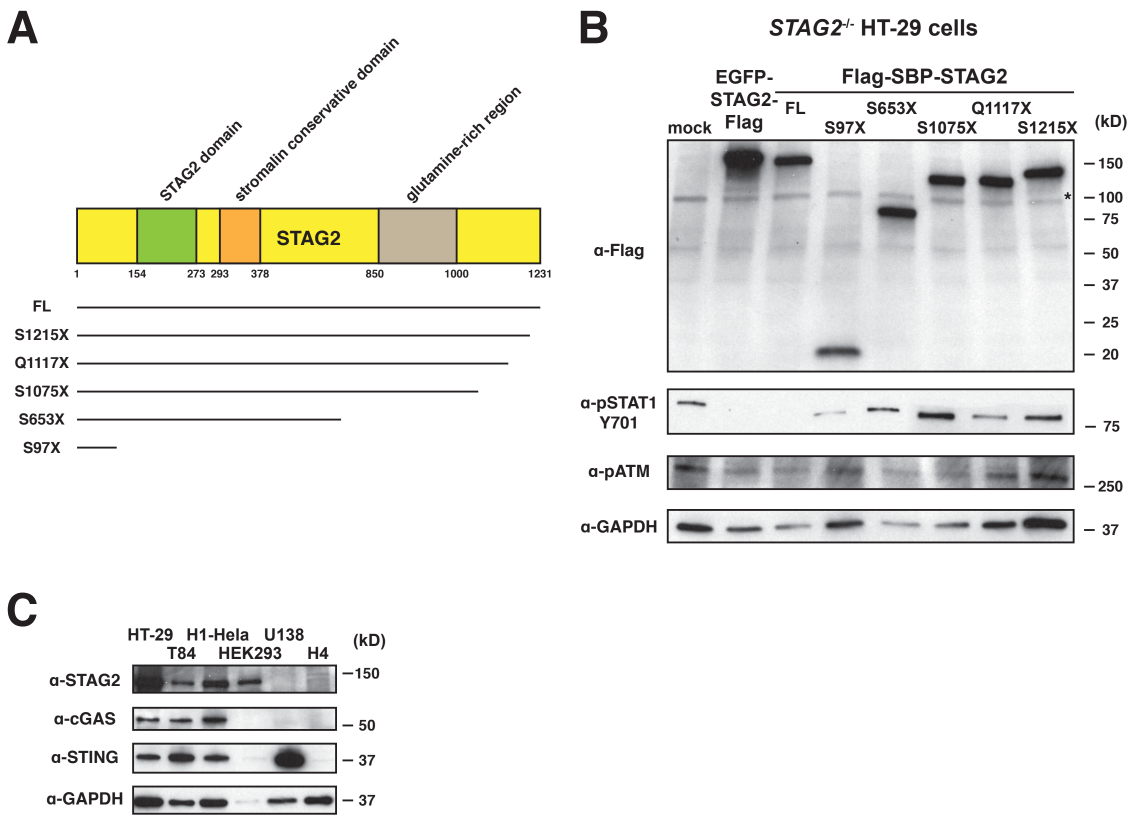

Supplementary Figure 9. STAG2 C-terminus is critical for suppressing DDR and IFN activation

(**A**) Schematics of STAG2 domains and mutants used in this study. (**B**) Lysates of *STAG2*$^{-/-}$ HT-29 cells reconstituted with indicated STAG2 mutants were analyzed by western blot using indicated antibodies (* represents a non-specific band). (**C**) Lysates of indicated cell lines (HT-29, T84: intestinal epithelial cells; H1-Hela: cervical epithelial cells; HEK293: embryonic fibroblast cells; U138, H4: glioblastoma cells) were analyzed by western blot for the levels of STAG2, cGAS, STING and GAPDH. For all figures, experiments were repeated at least three times in triplicates. Data are represented as mean ± SEM.

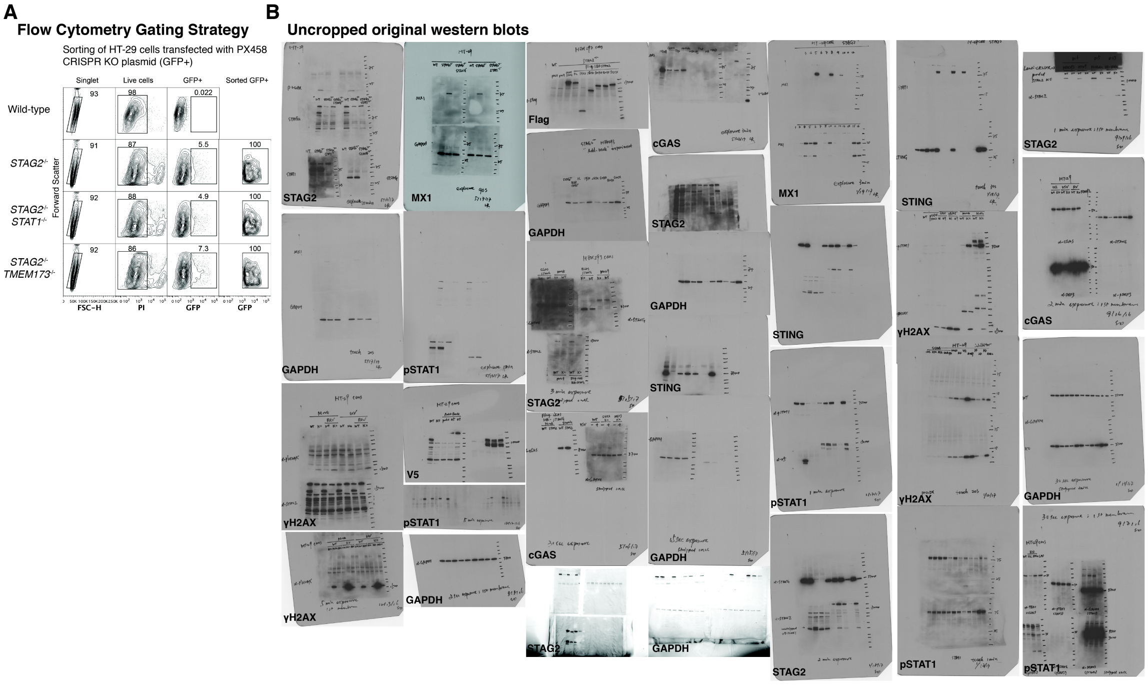

Supplementary Figure 10. Flow cytometry gating scheme and uncropped western blot images

(**A**) HT-29 cells were transfected with PX458 vector encoding Cas9 and sgRNA against *STAG2*, *STAT1* or *TMEM173* for 48 hr and subject to flow cytometry sorting using forward scatter to exclude cell debris and propidium iodide (PI) staining to exclude dead cells. GFP+ cells were selected and sorted into 96-well plates in a single-cell format. (**B**) Scans of films of western blots are provided.

Supplementary Table 1. Primer and siRNA information

QPCR and CRISPR Primer information:

RV NSP5	Probe	CY5/TCAAATGCAGTTAAGACAAATGCAGACGCT/IABRQSP
	Forward	CTGCTTCAAACGATCCACTCAC
	Reverse	TGAATCCATAGACACGCC
IFNB	Forward	ATGACCAACAAGTGTCTCCTCC
	Reverse	GGAATCCAAGCAAGTTGTAGCTC
IFNL1	Forward	CACATTGGCAGGTTCAAATCTCT
	Reverse	CCAGCGGACTCCTTTTTGG
IFNL3	Forward	TAAGAGGGCCAAAGATGCCTT
	Reverse	CTGGTCCAAGACATCCCCC
MX1	Forward	GTGGCTGAGAACAACCTGTG
	Reverse	GGCATCTGGTCACGATCCC
IAV	Forward	AAGACCAATCCTGTCACCTCTGA
	Reverse	CAAAGCGTCTACGCTGCAGTCC
CHIKV	Forward	AAGCTCCGCGTCCTTTACCAAG
	Reverse	CCAAATTGTCCTGGTCTTCCT
Ad5	Forward	GACATGACTTTCGAGGTCGATCCCATGGA
	Reverse	CCGGCTGAGAAGGGTGTGCGCAGGTA
SV40	Forward	TTAGCAATTCTGAAGGAAAGTCCTTG
	Reverse	AGCAGTGGTGGAATGCCTTTCATGAGG
VV	Forward	GGCAATGGATTCAGGGATATAC
	Reverse	ATTTATGAATAATCCGCCAGTTAC
DENV1	Forward	GCATATTGACGCTGGGARAGAC
	Reverse	TTCTGTGCCTGGAATGATGCTG
ZIKV	Forward	TTGGTCATGATACTGCTGATTGC
	Reverse	CCTTCCACAAAGTCCCTATTGC
HRV-A2	Forward	TCCTCCGGCCCCTGAAT
	Reverse	GAAACACGGACACCCAAAGTAGT
EV-D68	Forward	CACTGAACCAGAAGAAGCCA
	Reverse	CCAAAGCTGCTCTACTGAGAAA
VSV	Forward	GATAGTACCGGAGGATTGACGACTA
	Reverse	TCAAACCATCCGAGCCATTC
MAVS	Forward	GTGCCTACTAGCATGGTGCTC
	Reverse	GACCCAAGGCCCCTATTCT
TMEM173	Forward	AGCATTACAACAACCTGCTACG
	Reverse	GTTGGGGTCAGCCATACTCAG
GAPDH	Forward	GGAGCGAGATCCCTCCAAAAT
	Reverse	GGCTGTTGTCATACTTCTCATGG
RV NSP3	(+) Strand	CTCTCTGTTTCAATGACTCG
	(-) Strand	GTGGTGCGGTAGAAGTTG
STAG2	20nt-sgRNA	CTTCAGTCGTAGAGATCCAG
	Seq foward	GCAATTATTTGCGTGGTGTGC
	Seq reverse	GGTGGCTCTCCATTCTATTC
STAT1	20nt-sgRNA	GTGGAGCGGTCCCAGAACGG
	Seq foward	CCGTCCATCTCTTCGATATC

	Seq reverse	CCACAATATGTTCCAGAGAGTG
TMEM173	20nt-sgRNA	GCAGGCACTCAGCAGAACCA
	Seq foward	GAAAGGGGAACTGGGAG
	Seq reverse	AGTCACCTGGAGTGGATGT

Dharmacon SMARTpool siRNA information:

Non-targeting control: D-001810-10
MAVS: L-024237-00
TMEM173: L-024333-02
MB21D1: L-015607-02
IRF3: L-006875-00
BTRC: L-003463-00
PRKDC: L-005030-00
TREX1: L-013239-02
RAD50: L-005232-00
DDX41: L-010394-00
MYD88: L-004769-00
TICAM1: L-012833-00
IFI16: L-020004-00
AIM2: L-011951-00
ZBP1: L-014650-00

Supplementary Dataset 1. CRISPR-Cas9 screen results of RV infection

Column A: official gene symbol as listed in NCBI Gene Database

Column B: total number of hairpins (single-guide RNA) in the GeCKO library

Columns C-F: negative score (low 0 to high 1) as calculated by the MAGeCK algorithm

Column G: number of hairpins (single-guide RNA) not ranked in the top 35,000

Columns H-K: positive score (low 1 to high 0) as calculated by the MAGeCK algorithm

Column L: number of hairpins (single-guide RNA) ranked in the top 5,000

Supplementary Dataset 2. RNA-sequencing results of WT and *STAG2*$^{-/-}$ cells

Column A: official gene ID number as listed in NCBI Gene Database

Column B: official mRNA transcript number as listed in NCBI Nucleotide Database

Columns C and E: duplicate of Fragments Per Kilobase of transcript per Million mapped reads (FPKM) of *STAG2*$^{-/-}$ HT-29 cells on the BGI sequencing platform

Columns D and F: duplicate of FPKM reads of *STAG2*$^{-/-}$ HT-29 cells on the Illumina sequencing platform

Columns G and I: duplicate of FPKM reads of WT HT-29 cells on the BGI sequencing platform

Columns H and J: duplicate of FPKM reads of WT HT-29 cells on the Illumina sequencing platform

Column K: brief description of gene name listed in Column A

Uploaded as separate supplemental files

References

1 Bass, D. M. *et al.* Liposome-mediated transfection of intact viral particles reveals that plasma membrane penetration determines permissivity of tissue culture cells to rotavirus. *The Journal of clinical investigation* **90**, 2313-2320, doi:10.1172/JCI116119 (1992).

2 Urasawa, T., Urasawa, S. & Taniguchi, K. Sequential passages of human rotavirus in MA-104 cells. *Microbiology and immunology* **25**, 1025-1035 (1981).
