# Supplementary material for: STAG2 deficiency induces interferon responses via cGAS-STING pathway and restricts virus infection
Source: Nat Commun. 2018 Apr 16;9:1485. doi: 10.1038/s41467-018-03782-z (PMC5902600; doi:10.1038/s41467-018-03782-z)
Supplement: Supplementary file 3 — Description of Additional Supplementary Files [file 41467_2018_3782_MOESM3_ESM.pdf]

Description of Additional Supplementary Files

File Name: Supplementary Data 1
Description: CRISPR-Cas9 screen results of RV infection
Column A: official gene symbol as listed in NCBI Gene Database
Column B: total number of hairpins (single-guide RNA) in the GeCKO library
Columns C-F: negative score (low 0 to high 1) as calculated by the MAGeCK algorithm
Column G: number of hairpins (single-guide RNA) not ranked in the top 35,000
Columns H-K: positive score (low 1 to high 0) as calculated by the MAGeCK algorithm
Column L: number of hairpins (single-guide RNA) ranked in the top 5,000

File Name: Supplementary Data 2
Description: RNA-sequencing results of WT and STAG2-/- cells
Column A: official gene ID number as listed in NCBI Gene Database
Column B: official mRNA transcript number as listed in NCBI Nucleotide Database
Columns C and E: duplicate of Fragments Per Kilobase of transcript per Million mapped reads (FPKM) of STAG2-/- HT-29 cells on the BGI sequencing platform
Columns D and F: duplicate of FPKM reads of STAG2-/- HT-29 cells on the Illumina sequencing platform
Columns G and I: duplicate of FPKM reads of WT HT-29 cells on the BGI sequencing platform
Columns H and J: duplicate of FPKM reads of WT HT-29 cells on the Illumina sequencing platform
Column K: brief description of gene name listed in Column A
